# Dramatic Effect of Botox Injection in Disabling Chronic Migraine Secondary to Inoperable Cerebral Arteriovenous Malformation

**DOI:** 10.7759/cureus.53326

**Published:** 2024-01-31

**Authors:** Mohammed Mezaal, Malak Mohammed Abdulelah, Rawan Ahmed Mehanna

**Affiliations:** 1 Neurology, Dr. Sulaiman Al Habib Hospital, Dubai, ARE; 2 Surgery, Dubai Medical College, Dubai, ARE; 3 Family Medicine, Dubai Medical College, Dubai, ARE

**Keywords:** pregnancy, botox injection, chronic migraine, headache, cerebral arteriovenous malformation (camv), arteriovenous malformation (avm)

## Abstract

Arteriovenous malformation is a developmental anomaly of the vascular system characterized by arteriovenous shunt through a collection of tortuous vessels without intervening capillary bed. Brain arteriovenous malformations (AVMs) may cause hemorrhagic stroke, epilepsy, and chronic headache. Migraine with aura was reported in up to 58% of females with AVM.

A 23-year-old female presented with episodes of severe left-side headache for five months, throbbing in character with photophobia, phonophobia, and nausea. Brain MRI showed a large AVM in the left cerebellar hemisphere. She was diagnosed with grade six AVM, which is inoperable, and secondary migraine. Her migraine symptoms didn’t respond to oral medications. However, it responded dramatically to Botox injections. Seven days after Botox injection, her headache disappeared, and her well-being improved. Three years post-diagnosis and treatment, she got married, then three months later became pregnant. During pregnancy, she followed up with neurology, obstetrics, and gynecology. She was delivered by cesarean section to minimize the risk of intracranial hemorrhage and delivered without complications. The female patient in this case with migraine secondary to inoperable brain AVM treated with Botox; she got married and delivered by C-section without complications. This case raises the following important lessons: large AVMs can present with migraine only, and Botox has a dramatic effect on the treatment and the ability to have a safe pregnancy and delivery in large AVM cases.

## Introduction

Arteriovenous malformation known as AVM is a developmental anomaly of the vascular system characterized by arteriovenous shunting through a collection of tortuous vessels without an intervening capillary bed. AVMs can occur anywhere in the body; however, brain AVMs are of special concern because of the high risk of bleeding that can cause neurological damage. The cause of brain AVMs is unknown; however, it is possibly multifactorial; apparently both genetic mutation and angiogenic stimulation play roles in AVM development. Some believe that AVMs are congenital, while others advocate an angiopathic reaction, following either a cerebral ischemic or hemorrhagic event as a factor in their development [1,2]. AVM detection rate is 1.21/100,000 person-years (95% confidence interval {CI} 1.02-1.42) and the incidence of AVM-hemorrhage is 0.42/100,000 person-years (95% CI 0.32-0.55) [3].

Spetzler-Martin grading scale classifies operable cAVM into five following categories (I-V): lower grades I and II are a low risk when treating, grades III, IV, and V present greater risk, and grade VI is considered inoperable [4]. Brain AVMs may cause hemorrhagic stroke, epilepsy, chronic headache, or remain asymptomatic [5]. Headache with migraine features was reported in patients with cerebral AVM, both as migraine without aura and migraine with aura characteristics. In cases of females with AVMs, 58% were reported to have migraine with aura according to the International Classification of Headache Disorders, Third Edition (ICHD3) [6].

## Case presentation

**Figure 1:**
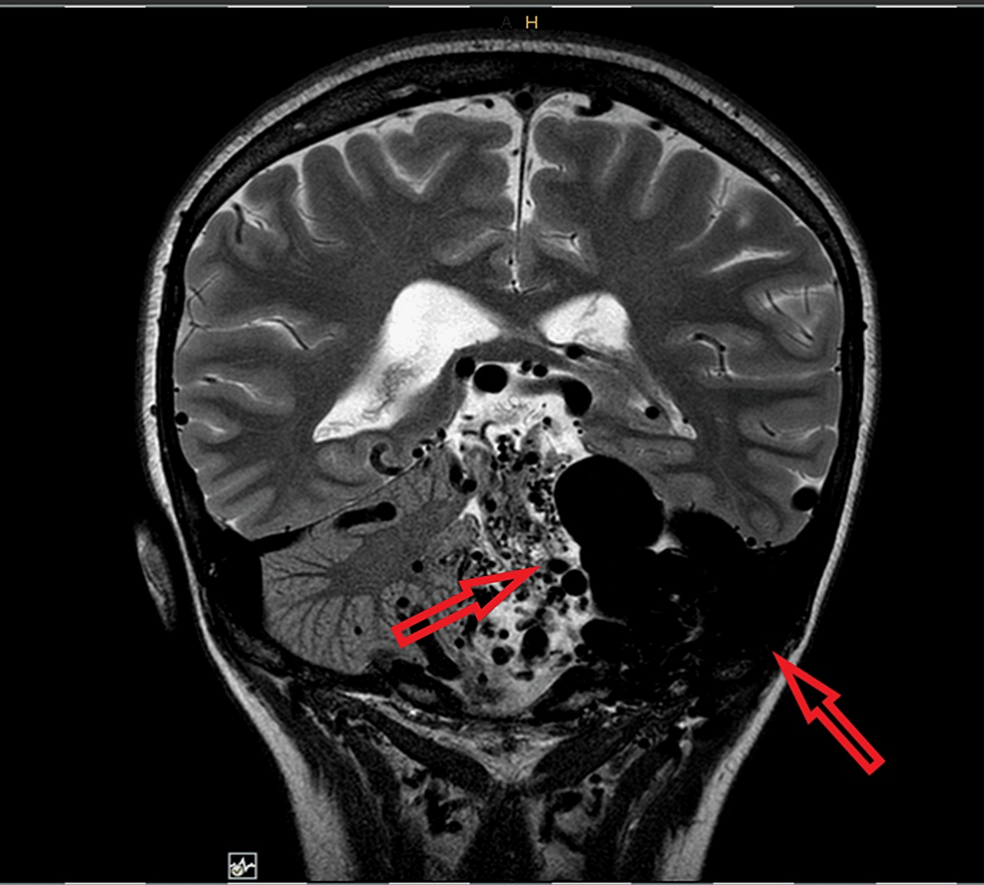
MRI coronal view showing extensive arteriovenous malformation with severe compressing phenomena. The image shows the involvement of posterior fossa structures and left occipital lobe. There is a narrowing of the fourth ventricle and dilation of the ventricular system.

A 23-year-old female patient presented with episodes of severe headache of five months duration on the left side which had increased in frequency for the last three months. Headache is throbbing in character and associated with photophobia, phonophobia, and nausea without vomiting. Each episode lasts for 8-48 hours with 18-20 days per month of headache of typical migraine features. No visual aura or other transient focal phenomena were noted. Neurological and general medical examinations were normal. Brain MRI without contrast was done, showing a large extensive arteriovenous, high-flow malformation identified with its epicenter in the left cerebellar hemisphere aspect of the posterior fossa. The malformation appears to be fed mainly by arteries originating from the posterior circulation (vertebral arteries, mainly the left one) and dilated meningeal artery (originates from the external carotid artery). The venous drainage is mostly into the vein of Galen and the straight venous sinus. There is a sizable aneurysmal vein that measures up to 2.3 cm in the axial plane at the left cerebellopontine angle. There are very severe compressive phenomena in the left aspect of the medulla oblongata, in the left cerebral hemisphere, left cerebellar peduncles, the pons, and the midbrain. There is a narrowing of the fourth ventricle and dilatation of the ventricular system. There is also some dilatation of the Meckel caves that is more prominent on the left (Figure 1).

**Figure 2:**
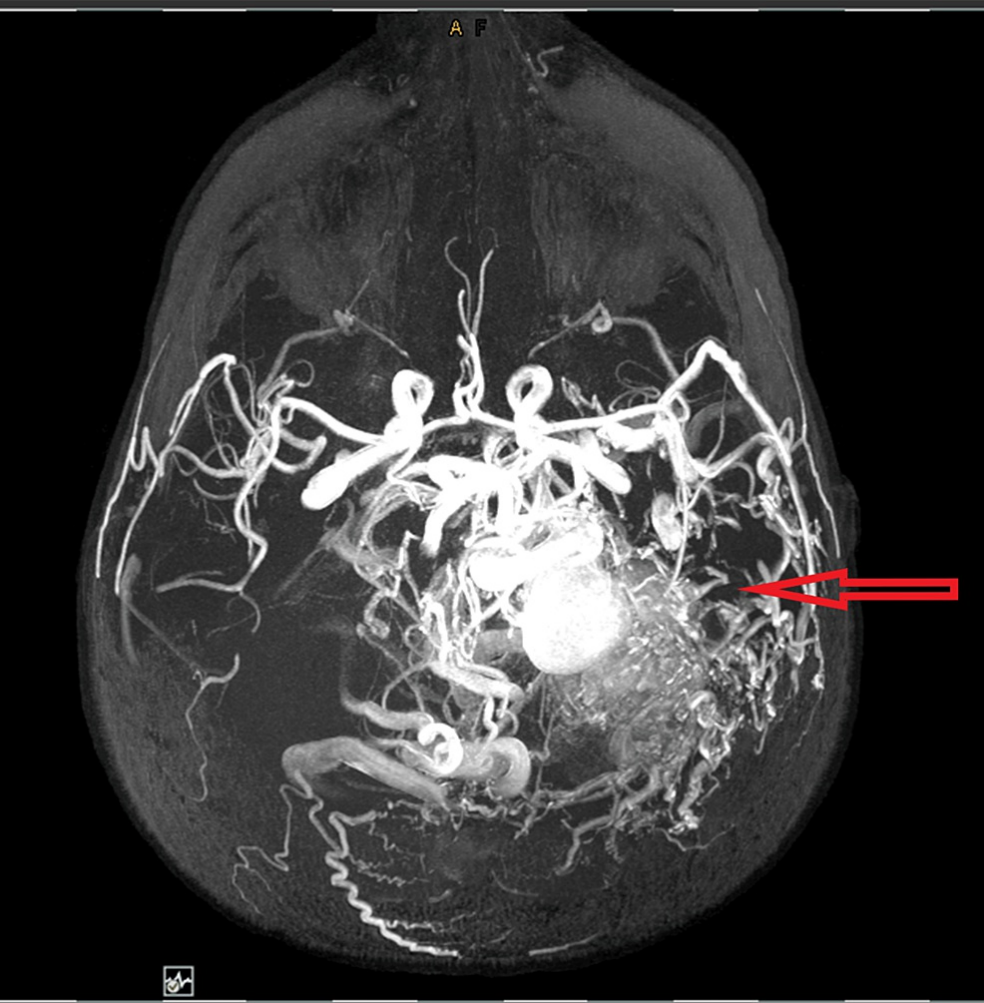
MRA/MRV scans show a large extensive arteriovenous high-flow malformation with a sizable aneurysmal vein that measures up to 2.3 cm.

She went for a second opinion abroad, but after doing and reviewing her MRI, magnetic resonance angiography (MRA), magnetic resonance venography (MRV), and conventional angiography findings, the diagnosis was confirmed as an extensive arteriovenous malformation and intervention was impossible with a high risk of mortality (Figure 2).

Her symptoms of migraine weren’t responding to oral medication after four months of trial. However, it responded dramatically to Botox injection 155 IU in 31 sites according to guidelines for Botox injection in chronic migraine, given at four to six months intervals [7].

After seven days of Botox injection, her headache disappeared almost completely, she is completely free from headaches, running a normal life, and her personality and mood improved. After three years she discussed a plan of marriage and the possibility of pregnancy, it was a very difficult decision with sociomedical aspects, especially in light of limited articles, if any, discussing the safety of pregnancy while harboring a giant inoperable brain AVM. Since she has been physically fit, active, working, and free of migraine symptoms for the past three years due to the effect of Botox injection, we agreed to the plan of marriage and discussed the risks of pregnancy and delivery in her case.

She got married, and after three months became pregnant. She followed up with neurology, obstetrics, and gynecology departments during pregnancy. In the third trimester of pregnancy, a decision of cesarean section was done to minimize the risk of intracranial hemorrhage during vaginal delivery. She delivered a healthy female baby and was discharged from the hospital after two days without complications. At present, she is running a normal life, and her baby is healthy and active. The MRI findings remained stable without changes in the size of AVM from 2019 until the last MRI done on August 17, 2023.

## Discussion

Brain arteriovenous malformations (AVM) are complex vascular lesions and it is associated with chronic headaches. An occipital location appears to increase the risk of concurrent migraine-like headaches in AVM patients [8]. Generally, in large AVM cases, the common presenting features include epilepsy symptoms or focal neurological deficits due to structural damage (intracerebral hemorrhage) and rarely migraine-like symptoms. However, when an arteriovenous malformation happens to be in the occipital lobe, patients would mainly present with migraine symptoms mostly with aura rather than seizures as the occipital lobe function is visual processing. Most cases of migraine in the occipital lobe are associated with visual aura. Pregnancy, delivery, and puerperium in patients with giant AVM are associated with a high risk of intracerebral hemorrhage [9].

We present a rare case of large inoperable brain AVM involving occipital lobe in a female patient of childbearing age who presented with a history of migraine headaches without aura; she got features of chronic migraine, not responding to oral medications like triptans group, topiramate, and NSAID but treated successfully with Botox injection. Botox injection was approved by the FDA in 2010 for the treatment of chronic migraine. Chronic migraine is defined as having unilateral headache on at least 15 days per month, with eight of these having migraine symptoms, for at least three months [10].

In our case, the presence of grade six AVM highly suggests secondary migraine rather than primary. However, taking into consideration her age and gender, primary migraine may also be present in this case, so the possibility of coexistence of both entities is there. The dramatic response to Botox suggests the option of this therapy in chronic migraine headache cases, whether primary or secondary to AVM, is effective and a treatment of choice; however, this observation needs more studies.

A literature review didn’t show any reports of migraine or chronic headache secondary to brain AVM treated successfully with Botox, and most cases reported were described the typical presentations where a patient had headache and seizures or other symptoms, which were commonly treated with analgesics or with surgical intervention in cases of operable AVMs. In addition to that, the majority of cases reported mentioned that, in cases of large brain AVM, developed intracerebral hemorrhage during pregnancy and/or delivery. But, in this study, she delivered by C-section to reduce the risk of aneurysm rupture without any complications [11,12].

## Conclusions

In this case, a young female patient presented with headaches with features of migraine without aura, and she was fulfilling the criteria of chronic migraine headache. Brain MRI and conventional angiogram showed large inoperable brain AVM. This case raised three lessons in cases of brain AVM not mentioned in literature. First, it was a large inoperable malformation in a very dangerous and eloquent area of the brain, and its only symptom was migraine headache without aura. The second and most important lesson is the dramatic effect of Botox injection in chronic migraine cases due to vascular anomalies. The second and most important lesson is the dramatic effect of Botox injection in chronic migraine cases due to vascular anomalies. Although it’s an uncommon modality of treatment in arteriovenous malformation, it showed a great symptomatic improvement. The third lesson is that this case poses a great challenge to the concept that large inoperable brain AVM is associated with a high risk of intracerebral hemorrhage during pregnancy, delivery, and puerperium.
